# A case series: Three cases of Morvan’s syndrome as a rare autoimmune disorder with anti-Caspr2 antibody

**DOI:** 10.1097/MD.0000000000040159

**Published:** 2024-10-18

**Authors:** Dan Ma, Qiong Xiang, Zhengbo Mo, Qilian Du, Yanqing Tang, Shasha Mei, Enfeng Song

**Affiliations:** a Clinical Department of Traditional Chinese Medicine, Hubei University of Chinese Medicine, Wuhan, China; b Department of Traditional Chinese Medicine, Renmin Hospital of Wuhan University, Wuhan, China.

**Keywords:** anti-Caspr2 antibody, case series, Morvan’s syndrome, neuromyotonia, Traditional Chinese Medicine

## Abstract

**Rationale:**

Morvan syndrome (MoS) is an uncommon male-dominant autoimmune disorder marked by peripherally innervated hyperexcitability, autonomic disturbances, and encephalopathic encephalopathy, frequently with mass complaints manifesting as neuromyotonia (involuntary jerking, twitching, and stiffening of muscles), myotonia, neuropathic pain, hyperhidrosis, severe constipation, and severe sleep disturbances accompanied by dream reenactments, agrypnia agitation, and delusions, associated with autoantibodies to voltage-gated and potassium channel complexes such as anti-contactin–associated protein-like 2 (Caspr2) antibody. All this misery can be very disabling and even life-threatening. Reported cases show an unforeseeable outcome, with fatalities occurring even in those who initially responded. It has been reported that patients have reacted to immunologic therapies—corticosteroids, intravenous immunoglobulins, plasma exchanges, azathioprine, cyclophosphamide, rituximab, or carbamazepine, gabapentin, and clonazepam. However, no long-term effective cure has yet been found for this condition. Clinicians and researchers increasingly emphasize alternative and complementary medicine, with a growing trend toward traditional Chinese medicine (TCM).

**Patient concerns:**

Following glucocorticoid therapy, all 3 patients experienced a recurrence of the disease. Patients 1 and 2 observed symptomatic relief after intravenous immunoglobulin administration; however, upon discontinuation of the treatment, their conditions relapsed and worsened compared with the previous state.

**Diagnoses:**

The 3 patients were definitively diagnosed with serum Caspr2-positive MoS, accompanied by a constellation of neurological manifestations.

**Interventions:**

The 3 patients were treated under the guidance of TCM theory. According to the principles of TCM, the patients were characterized by the deficiency of *Yin*, so the prescriptions were as follows: Shaoyao-Gancao decoction combined with Sanjia-Fumai decoction.

**Outcomes:**

After the application of TCM, there was a reversal of neuropsychiatric manifestations such as unintentional rippling, jerking, muscle stiffness, myokymia, hyperhidrosis, and extreme constipation. Patients’ quality of life improved significantly; to date, they have achieved Karnofsky Performance Status scores of 100, and the anti-Caspr2 antibody result in case 2 dropped from 1:32 to normal.

**Lessons:**

We first report the effective treatment of the MoS case series with TCM as complementary and alternative medicine.

## 1. Introduction

Morvan’s syndrome (MoS) is a rare cluster of neurological symptoms that includes a group from clinical syndromes marked by hyperexcitability of peripheral nerves, autonomous dysfunction, and encephalopathy, often with severe insomnia, ranging from mild to malignant forms characterized by total sleep loss, accompanied by dream enactment and apnea excitation associated with autoantibodies to voltage-gated and potassium channel (VGKC) complexes such as anti-contactin-associated protein-like 2 (Caspr2) antibody. These disorders can be profoundly disabling and even life-threatening. Close cooperation between specialists and family members is required to care for these patients appropriately. Some patients have been reported to have a response to immunotherapy-corticosteroids,^[1,2]^ intravenous immunoglobulins (IVIg),^[3]^ plasma exchange,^[4,5]^ azathioprine,^[6]^ cyclophosphamide,^[7]^ rituximab,^[8,9]^ or carbamazepine, gabapentin, and clonazepam^[10]^; however, some relapse quickly with steroids and immunotherapy.^[8]^ No long-term effective cure has been found for this condition. Cases reported to date show an unpredictable prognosis, with deaths occurring even in those who initially respond.^[11]^ Clinicians and researchers are turning to complementary and alternative medicine, particularly Traditional Chinese Medicine (TCM).

We treated 3 anti-Caspr2 antibody-positive patients with MoS who suffered from the disorders and had a poor health-related quality of life (HRQoL) with TCM. HRQoL was assessed using the Karnofsky Performance Status (KPS). After the application of TCM, there was a reversal of neuropsychiatric manifestations such as unintentional rippling, jerking, muscle stiffness, myokymia, hyperhidrosis, and extreme constipation. Patients’ quality of life improved significantly; to date, they have achieved KPS scores of 100, and the anti-Caspr2 antibody result in case 2 dropped from 1:32 to normal. We first report the effective treatment of the MoS case series with TCM as complementary and alternative medicine.

## 2. Methods

### 2.1. Information about the patients, clinical findings, and timeline

**Figure 1.**
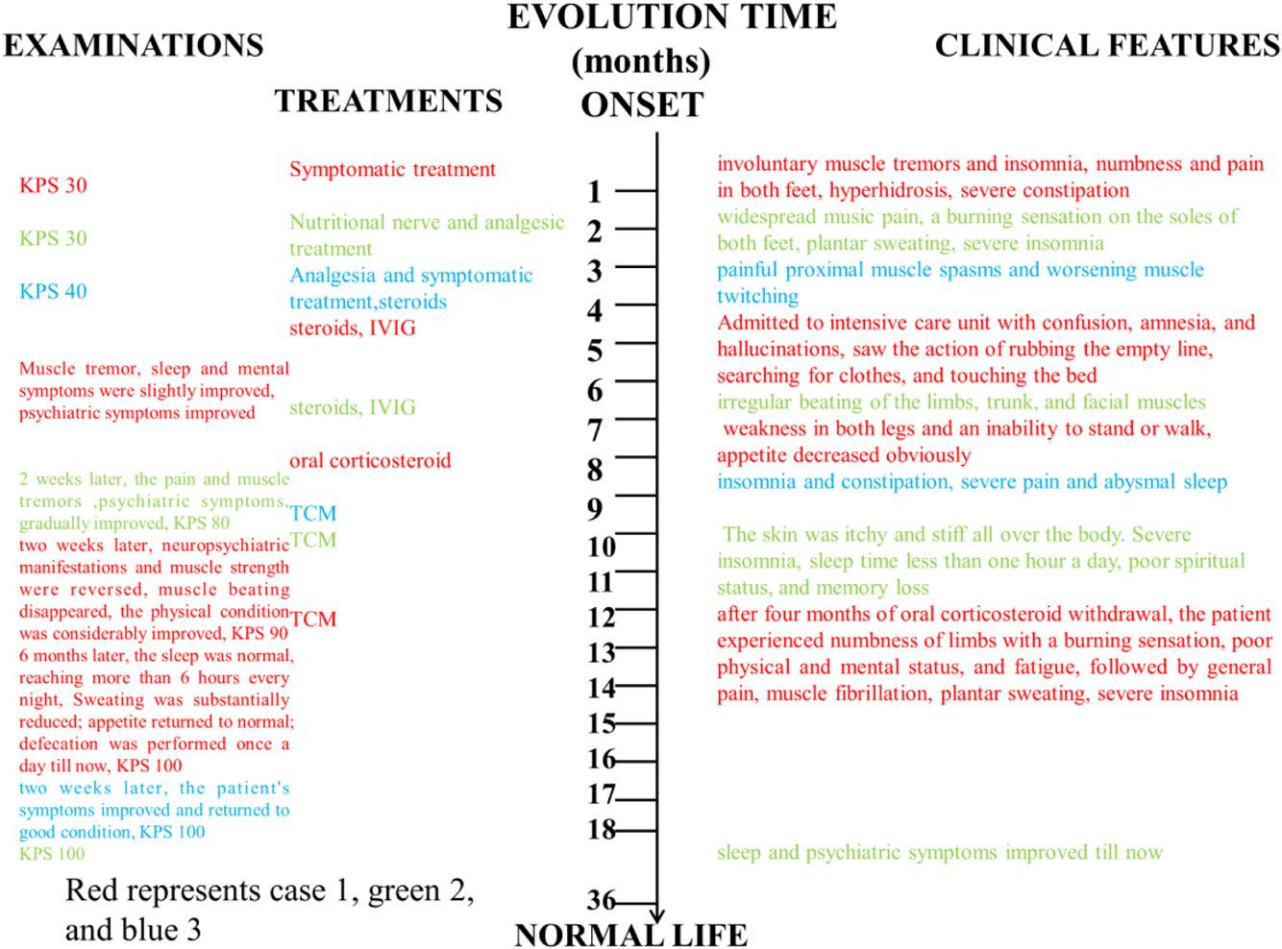
The timeline of appearance and treatments and examinations of these symptoms. TCM = Traditional Chinese Medicine; KPS = Karnofsky Performance Status; IVIg = immunoglobulin.

Our study results have been reported by the CARE guidelines.^[12]^ Here, we describe 3 cases of MoS patients who have presented to our department since July 2015. The most essential characteristics of the cases described in this report are summarized in Table 1. All 3 patients were definitively diagnosed with MoS. The timeline of appearance and examinations of these symptoms is illustrated in Figure 1.

**Table 1:** Clinical details and detailed medication history of the 3 patients at admission in brief.

Case no.	1	2	3
Age	65	59	52
Gender	Male	Male	Male
Admission	Wheelchair	Wheelchair	Helped walking
Chief complaint	Had involuntary muscle tremors and insomnia for over 3 mo, accompanied by numbness and pain in both feet, hyperhidrosis, and severe constipation	Had muscle pain for more than 2 yr, a burning sensation on the soles of both feet, accompanied by plantar sweating, severe insomnia for more than 1 yr	Had 2 mo of painful proximal muscle spasms and worsening muscle twitching for 10 d
Myokymia	The frequency of muscle tremors was fast, up to 150 times per minute; it was almost impossible to live normally	Irregular beating of the limbs, trunk, and facial muscles	Experienced involuntary beating of limbs, chest, and abdomen
Neuropathic pain	The waist and leg pain	Gradual onset of calf pain, thigh pain, and progressive worsening of pain	Limb soreness even affected sleep
Hallucination	Sometimes, he was clear and confused; sometimes, he had confusion, amnesia, and hallucinations, and occasionally, he saw the action of rubbing the empty line, searching for clothes, and touching the bed	Sometimes forgetful and confused	Occasionally appeared
Sleep disorder	Severe insomnia, sleeping for up to 2 h daily, easy to wake up from much dreaming	Severe insomnia, sleep time less than 1 h/d, poor spiritual status, and memory loss	Severe insomnia
Autonomic disturbances	He had severe constipation and defecation once every 7–10 d. He needed laxative drugs to defecate and urinate normally. He was weak and sallow, and his physical strength and weight decreased markedly. The whole body sweated more and worse at night	He had severe constipation and abdominal distension; he only pooped about once every 10 d; he did not defecate, relied on enemas, and exhibited oliguria, aggravated sweating at night	He had severe constipation
General condition	There was weakness in both legs and an inability to stand or walk. His appetite decreased obviously; little liquid food was taken	The skin was itchy and stiff all over the body. He could not walk more than 20 m away at most and experienced fatigue, difficulty walking independently, and inability to hold things firmly in his hands	Fatigue was evident
Detailed medication history	Zyprexa (olanzapine), Sid (Tandospirone citrate tablets), methyl prednisone, Immune globulin, etc	Mecobalamin tablet, etoricoxib, vitamin B1, folic acid, diclofenac sodium, gabapentin capsule, methyl prednisone, Immune globulin, etc	Fenbid, tramadol, pregabalin, sustained-release lofencodeine tablets, gabapentin capsule, mecobalamin tablet, clonazepam, dexamethasone, methyl prednisone, etc

Patient 1 had involuntary muscle tremors (Video 1, Supplemental Digital Content, http://links.lww.com/MD/N748) and insomnia for over 3 months, accompanied by numbness and pain in both feet, hyperhidrosis, and severe constipation. He had no family medical or tobacco history and was an alcoholic with a 40-year history.

Patient 2 was a 57-year-old man with a preexisting diagnosis of lumbar disc herniation and a long chronic history of lumbago. He had a record including smoking for approximately 8 years, a pack every 2 days, and drinking alcohol for 30 years. Unfortunately, he suffered from general pain, especially back pain, accompanied by the feeling of stepping on cotton from February 2020. Then came the experience of a plantar burning sensation in both feet, accompanied by plantar sweating, the gradual appearance of calf pain, thigh pain, and massive sweating of the whole body. Sweating at night increased, followed by pain in the limbs and the entire body, muscle fasciculation, irregular beating of limbs, trunk, and facial muscles, itching of the whole body, stiff skin, difficulty in walking independently, severe insomnia, poor mental performance, and memory loss. After more than a year of immunoglobulin and steroid therapy, the patient’s muscle fibrillation, pain, and sweating improved, but severe discomfort relapsed after 4 months of withdrawal.

Patient 3 is an ex-smoker with a 25-year history and preexisting lumbar disc herniation and nephritic syndrome diagnoses. He suffered 2 months of painful proximal muscle spasms and ten days of worsened muscle twitching. He also experienced involuntary beating of limbs, chest, and abdomen, followed by insomnia and constipation.

### 2.2. Informed consent and ethics clearance

Written informed consent was obtained from the patients on a free and voluntary basis, and clinical data were fully anonymized. The Clinical Research Ethics Committee approved the study protocols, Renmin Hospital of Wuhan University (WDRY2023-K111). This research was conducted following the Declaration of Helsinki and its subsequent modifications.

### 2.3. Diagnostic assessment and therapeutic intervention

The key findings confirming the diagnosis of MoS and the clinical details of the 3 patients are briefly summarized in Table 2. The relevant examination data included anti-Caspr2 Abs and anti-leucine-rich glioma inactivated 1 (LGI1) Abs results in serum and cerebrospinal fluid, electromyography (EMG), and magnetic resonance imaging (MRI) to support the diagnosis of MoS. General examination and laboratory tests, including those for hepatitis B and C, human immunodeficiency virus (HIV), antinuclear autoantibodies, anti-neutrophil cytoplasm, anti-acetylcholine receptor antibodies, tumor markers, liver and kidney function, urine routine, blood routine, electrocardiogram were within the normal ranges. Brain MRI, EMG, and clinical signs and symptoms are summarized in Table 2. HRQoL was assessed using the KPS, and the distribution of the absolute differences in KPS scores at the first visit to follow-up evaluation at 6-month intervals after that were recorded.

**Table 2:** Examination results of the 3 patients.

Case no.	1	2	3
Anti- Caspr2 Abs			
Serum	1:32	1:32	1:10
CSF	Normal	NA	Normal
Anti-LGI1 Abs			
Serum	1:10	Seronegative	1:10
CSF	NA	NA	NA
Brain MRI	+*	+†	+‡
EMG Pnh	+§	+§	+∥
Thymoma on thorax/neck CT	Normal	Normal	Normal
KPS score			
On admission	30	30	40
After treatment of TCM (months)			
1	80	90	80
2	90	90	90
6	90	90	90
12	90	90	100
24	100	100	100
36	100	100	100

CASPR 2 = contactin-associated protein-like 2, CSF = cerebrospinal fluid, CT = computed tomography, EMG = electromyography, KPS = Karnofsky Performance Status, LGI1 = leucine-rich glioma inactivated 1, MRI = magnetic resonance imaging, NA = not available, Pnh = features suggestive of peripheral nerve hyperexcitability;, TCM = Traditional Chinese Medicine.

* No abnormal empty flow signal was found in the skull. The results indicated the following: ① multiple lacunar cerebral infarctions occurred in the brain stem, bilateral basal ganglia, radiative coronal area, removable center, and subfrontal parietal cortex; ② leukoencephalopathy, cerebral shrinkage.

† Lateral removable ischemia in the left subfrontal cortex; senile brain.

‡ Multiple ischemic lesions in the bilateral subfrontal, parietal cortex.

§ Neurogenic injury of lower extremities (a large amount of flutter wave potential can be seen).

∥ The peripheral nerve conduction detected was within the range of positive perception. Neurogenic lesions were observed in the right triceps brachii, the biceps brachii, the right deltoid, the right gastrocnemius, the right first dorsal interosseous, the right rectus femoris, the right tibialis anterior, and the left biceps brachii were within the normal range.

¶Electrophysiological manifestations of the bilateral peroneal nerve, tibial nerve, sural nerve, and femoral posterior cutaneous nerve injury. Abnormal F wave of the lower extremity.

The diagnosis of MoS requires at least 4 of the following: myokymia or nerve myotonia, dysautonomia, rigorous sleep disturbances, and fluctuant encephalopathy with prominent hallucinations.^[10,13]^ EMG shows that the voluntary motor unit action potentials fire as doublets or triplets, and brain MRI is usually not abnormal, with EEG either normal or showing scattered slowing. Most patients have anti-Caspr2 Abs in their serum.^[14]^ The pathophysiology is unclear; malignancy (most commonly thymoma,^[10]^ lung cancer^[15]^), clinical or subclinical myasthenia, familial Mediterranean fever gene mutations, and panuveitis have been associated with MoS.^[16,17]^

The clinical presentation of the patients was consistent with the above diagnostic criteria. A diagnosis of MoS was considered based on the patient’s history, EMG, MRI, serum antibody results, and medication history.

### 2.4. Treatment and follow-up

Details of medication history are shown as follows. Symptoms of case 1 did not improve with the treatment of corticosteroids and immunotherapy at the local hospital; he then came to our department for comprehensive care. The patient was treated with TCM from July 12, 2015 to September 5, 2015. At follow-up, the neuropsychiatric manifestations and normal muscle strength were reversed. The patient said that muscle beating disappeared, the physical condition was considerably improved, the life could take care of itself, the sleep was normal, reaching more than 6 hours every night, and it was easy to fall asleep. Sweating was substantially reduced; appetite returned to normal; defecation was performed once a day, urination was expected, and there were no apparent other discomfort symptoms; up to now, he engages in agricultural work at home independently.

Patient 2 had neuropathic pain in both legs, pruritus, hyperhidrosis, and myokymia. No improvement of myokymia was observed after self-administration of mecobalamin and etoricoxib. He received vitamin B1, folic acid neurotrophic therapy, diclofenac sodium, and gabapentin capsules for pain relief at the local hospital. However, he gained little. Then received a remote consultation from specialists in Beijing and was diagnosed with MoS; the patient was given treatment with intravenous immunoglobulin and high-dose steroids. Steroids were gradually stopped after 1 year of oral administration, then the pain and muscle tremors gradually improved. However, after 4 months of drug withdrawal, the patient experienced numbness of limbs with a burning sensation, poor physical and mental status, and fatigue, followed by general pain, muscle fibrillation, plantar sweating, severe insomnia, <3 hours of sleep per day, and constipation. Luckily, with the treatment of TCM, the patient’s muscle fibrillation, sweating, insomnia, constipation, and other discomfort were considerably improved, and he could walk and work independently.

Patient 3 was given oral administration of fenbid, tramadol, pregabalin, and lofencodeine sustained-release tablets at the indigenous hospital. However, after drug discontinuation, the patient still had obvious neuropathic pain, myokymia, insomnia, and constipation. After TCM treatment, the patient’s symptoms improved, and he returned to good condition.

Three patients relapsed after glucocorticoid treatment, and cases 1 and 2 had severe symptoms after IVIg, and they would seek help from TCM. All 3 patients were treated under the guidance of TCM theory. According to the theory of TCM, the patients were characterized by the deficiency of *Yin*, so the prescriptions were as follows: Shaoyao-Gancao decoction combined with Sanjia-Fumai decoction; Its efficacy is: nourishing *Yin* and soothing pain and stopping spasticity; Prescription: White *paeoniae* root 30 g, fried *glycyrrhizae* 10 g, *raw rehmannia radix* 10 g, *ophiopogon* 10 g, *hemp seed* 20 g, *calcined keel* 30 g, *calcined oyster* 30 g, *floating wheat* 30 g, *Gastrodia elata* 10 g, *Rhynchophylla* 20 g, 7 doses for a course of treatment, 1 dose per day, boiling these herbs into 400 mL soup with water, each dose is divided into the morning and evening half an hour after meals.

## 3. Results

After 2 courses of medication, the patients’ muscle fibrillation, sweating, insomnia, constipation, and other discomfort were considerably improved, and they could walk and work independently. Their KPS scores are summarized in Table 2, showing marked variability from poor to almost normal after TCM treatment. Patients 1 and 2 (KPS 30) came to the hospital in a wheelchair, and patient 3 (KPS 40) was helped in the consulting room. Their HRQoL was lousy but improved considerably. In addition, in patient 2, the anti-Caspr 2 antibody result returned to normal from 1:32. At the most recent follow-up, the 3 patients had relatively high KPS scores and enjoyed their independence, and no more obvious discomfort symptoms occurred.

## 4. Discussion and conclusions

We first report the effective treatment of the MoS case series with TCM as complementary and alternative medicine, that patient 2’s antibodies switched from positive to negative, and the quality of life returned to normal in 3 patients, all of whom had a high quality of life during the follow-up period. After applying TCM, the patients’ muscle tremors, pain, heavy sweating, severe insomnia,^[18]^ serious constipation, and other uncomfortable symptoms disappeared. This is consistent with previous clinical studies on TCM relieving insomnia,^[18]^ constipation, hyperhidrosis,^[19]^ and muscle fibrillation.^[20–23]^

TCM has benefited twenty percent of the world population by treating various diseases. The ancient holistic approach of Chinese practitioners suggests that a multitude of events are crucial to returning a patient to a healthy condition. It has a long history of being used in treating neuro-related diseases. It is a main form of complementary and alternative medicine that provides a possibility for central nervous system disease management and has been applied in large numbers of patients with such disorders—involuntary shaking, jerking, stiffening of muscles, or myokymia. MoS belongs to the “tremor” category, introduced more than 600 years ago, and has a complete etiology and pathogenesis description in TCM. The basic pathogenesis is *Yin* deficiency, a concept in TCM that refers to an imbalance or depletion of the *Yin* energy within the body. *Yin* represents the nourishing, cooling, and moistening aspects of our body. *Yin* deficiency means an insufficiency or imbalance in these nourishing and moistening aspects. *Yin* deficiency may result from various factors such as aging, chronic diseases, excessive physical or mental activities, poor diet, and prolonged stress. Based on the TCM theory, if a patient has sweating, constipation, insomnia, dry mouth, thin pulse, and red tongue without furred, we may judge *Yin* deficiency symptoms and give the corresponding TCM treatment—all 3 patients presented with *Yin* deficiency. We chose the combination of the prescription “Peony Licorice Decoction” and “Sanjia Fumai Decoction” to nourish *Yin*, raise blood effectiveness, and soothe the nerve. TCM has a relatively complete theory and rich experience in treating flutter syndrome.

“Peony Licorice Decoction” is from “Treatise on Febrile Diseases” (*Shang Han Lun*), also named Shaoyao-Gancao Decoction, a combination of *Radix Paeoniae* and *Glycyrrhizae Uralensis*. The prescription has multicomponent, multitarget, and multichannel anti-spasmodic, anti-inflammatory, and analgesic aspects and can treat musculoskeletal system pain.^[21]^ Peony Licorice Decoction may be due to the presence of these ingredients, so it has a good relief for the pain and other discomfort caused by MoS. “Sanjia Fumai Decoction” is from “Wen Disease Strip Discrimination,” mainly used for treating hand and foot peristalsis, palpitations, convulsions, dry mouth, dry tongue, and weak pulse. In modern clinical research, “Sanjia Fumai Decoction” has been reported in the treatment of insomnia,^[18,20]^ hyperhidrosis, tremor syndrome,^[20,24]^ hypertension,^[25]^ vertigo,^[24]^ and other neurological diseases. MoS is a complex and multi-symptom disease similar to the indications of “Sanjia-Fumai decoction” and “Peony Licorice Decoction.” In future studies, further experiments are needed to elucidate the specific mechanisms.

MoS is a rare autoimmune disorder that predominantly affects middle-aged and older men, with a male to female-presentation ratio of (9–19):1 in adult patients and a median age of onset of 57 years.^[10,26]^ Patients 1, 2, and 3 in our article were all around this age and were all male. As a complex autoimmune disease with disturbances of the central nervous system (CNS) and hyperactivity of the autonomic and peripheral nervous systems (PNS), the clinical symptoms of MoS mainly include 3 aspects: ① CNS involvement manifesting as encephalopathy, mental confusion, disorientation, temporal and spacial disturbances, severe sleeplessness, complex hallucinations, delirium, and restlessness; ② Autonomic dysfunction. It includes the syndrome of inadequate anti-diuretic hormone secretion, cardiac arrhythmia, hypertension, weight loss, hyperhidrosis, palmoplantar erythema; ③ peripheral nerve hyperexcitability results in neuropathic pain, areflexia, stocking-like sensory loss, and continuous muscle fiber activity (neuromyotonia), which manifests clinically as myokymia.^[4]^ For years, light hypersensitivity and infection, as well as metal intoxication and mercury exposure, were considered possible causes.^[9]^ It was later recognized that patients with MoS have VGKCs complex antibodies, primarily directed against Caspr2. VGKCs are expressed on the membrane of neurons in both the CNS and the PNS, where they are involved in repolarization following an action potential. Research on specific antigenic components of the VGKCs antibody, such as Caspr2 and LGI1 autoantibodies, has shown some correlation between clinical manifestations and the topographic distribution of these antibodies within the CNS. In addition, the literature has also reported anti-VGKCs antibody-negative cases of MoS. Observation of clinical case series of Caspr2-related MoS,^[27]^ another retrospective case series described 14 patients with Caspr2 antibody–related MoS had various neurologic manifestations.^[28]^ An up-to-date cohort study identified 164 patients with Caspr2 autoantibodies; over 90% had only Caspr2, and just 15 (9.1%) had combined Caspr2 and LGI1 autoantibodies. A small proportion of patients with double-positive LGI1/Caspr2 autoantibodies (15/164) were significantly more likely to have developed myoclonus, tremulousness, mixed movement disorders, MoS, and associated underlying tumors.^[29]^ Anti-Caspr2 patients have variable phenotypes with central and peripheral symptoms, and anti-LGI1 encephalitis patients have largely limbic features.

Caspr2 is an axonal transmembrane protein of neurexin found in the juxta paranodal region (node of Ranvier), hippocampus, and cerebellum.^[30]^ Caspr2 associates with contactin-2 to form an axonal transmembrane protein complex in the juxtaparanodal region of myelinated axons. This Caspr2-contactin-2 complex is highly expressed in CNS inhibitory neurons and controls axonal excitability by preventing repetitive firing and stabilizing conduction through the nodes of Ranvier. Caspr2 antibodies target inhibitory interneurons in the hippocampal region, disrupting the interaction between Caspr2 and contactin-2 and altering gephyrin accumulation in the postsynaptic area. Because the Caspr2 protein is expressed in both CNS and PNS axons, these patients may develop a syndrome of associated peripheral nerve hyperexcitability with neuromyotonia, fasciculations, and spasms that may precede encephalitic symptoms. Most reports of Caspr2 autoimmunity consist of clinically selected populations of patients with MoS, refractory focal epilepsy, or pain syndromes. The majority of patients with demonstrated Caspr2 antibodies were male (89%–90%),^[31]^ which is in line with the male-dominated MoS, but the reasons are currently unknown.^[32]^ In our article, the 3 patients were anti-Caspr2 Abs positive, as was anti-LGI1 Abs in patients 1 and 3.

Neurons secrete LGI1 and are not a structural constituent of a receptor or ion canal. LGI1 antibodies are thought to enhance neuronal excitability, which can manifest as epileptic seizures. The great majority of patients with anti-LGI1 encephalitis present with clinical symptoms of limbic encephalitis: temporal lobe epileptic convulsions, memory loss, and behavioral alterations. The seizures may be of a focal, tonic, clonic, generalized, or facial-brachial dystonic type. Other characteristic symptoms these patients may develop are sleep disturbances (especially sleep insomnia) and autonomic disturbances. Treatment of patients with LGI1 antibody encephalitis consists of Methylene Predisolone, IVIg, and plasma exchange.

There is no established management for MoS. Various immunosuppressive approaches have been used with inconsistent results, such as immunotherapy-corticosteroids, IVIg, plasma exchange, azathioprine, cyclophosphamide, rituximab, and other immunosuppressant agents. One case report stated that plasma exchange was effective after glucocorticoid treatment of a relapse. Still, another reported that both the peripheral and central symptoms improved remarkably with corticosteroid therapy, while plasma exchange, formerly considered the first-line treatment, was ineffective. Patients reportedly had a heterogeneous reaction to the immunomodulation. Patients 1 and 2 were treated with steroids and immune globulin with suboptimal results, and Patient 3 was not treated with immune globulin. Their symptoms were significantly improved after treatment with TCM, and they have not relapsed.

Our case series report provides a new possible option for treating MoS. In this way, patients with rare diseases have more hope and choices. However, there are still several limitations in the article, such as the number of cases is too small, with only 3 cases reported, more clinical cases need to be collected, and a multicenter randomized controlled study should be designed to observe the clinical efficacy of TCM to support the idea with high-level evidence. Since it is a rare disease that takes a long time to collect cases, we want to inspire new ideas for clinicians and patients through this paper. In addition, the mechanism of TCM in treating MoS needs to be further investigated to clarify which TCM components play a significant role, which needs to be elucidated by further experimental studies.

### 4.1. Patient’s perspective

We sought the guidance of TCM after failing to control the condition with immunotherapy - corticosteroids and intravenous immunoglobulin. Given that the symptoms were complicated, we tried to obtain TCM decoction according to the theory of Chinese herbal medicine. After 2 weeks of treatment, the patients’ muscle fibrillation, sweating, insomnia, constipation, and other discomfort were considerably improved. This encouraging turnaround gave us great confidence in the current treatment schedule. The medical caregivers were patient and attentive. Throughout the therapy period, we were pleased to see their progress.

## Acknowledgments

We thank the patients for participating and providing detailed case data. We thank the Construction Project supported by the National Famous Old Chinese Medicine Experts Inheritance Studio (National Chinese Medicine Human Education Letter [2022] No. 75) for funding this research. We also thank AJE for providing us with language editing services.

## Author contributions

**Conceptualization:** Enfeng Song.

**Data curation:** Dan Ma.

**Investigation:** Qilian Du, Yanqing Tang.

**Methodology:** Enfeng Song, Zhengbo Mo.

**Supervision:** Zhengbo Mo.

**Software:** Qilian Du.

**Visualization:** Qiong Xiang.

**Writing – review & editing:** Enfeng Song, Dan Ma, Qiong Xiang, Shasha Mei.

**Writing – original draft:** Dan Ma.

## Supplementary Material


